# Corticomuscular Coherence in Post-Stroke Motor Function and Recovery

**DOI:** 10.3390/brainsci16070689

**Published:** 2026-06-30

**Authors:** Rachana Gangwani, Jasper I. Mark, Sabrina Zadrozny, Jessica M. Cassidy

**Affiliations:** 1 Human Movement Science Curriculum, University of North Carolina at Chapel Hill, Chapel Hill, NC 27514, USA; rachana.gangwani@jefferson.edu (R.G.); jaspermark13@gmail.com (J.I.M.); 2 Department of Health Sciences, University of North Carolina at Chapel Hill, 321 S. Columbia Street, Chapel Hill, NC 27514, USA; 3 Frank Porter Graham Child Development Institute, University of North Carolina at Chapel Hill, Chapel Hill, NC 27514, USA; sabrinaz@unc.edu

**Keywords:** stroke, electroencephalography, motor, recovery, electromyography, rehabilitation

## Abstract

**Highlights:**

**What are the main findings?**

- Individuals with stroke demonstrated reduced brain–muscle functional connectivity (corticomuscular coherence) between the supplementary motor area (SMA) and affected first dorsal interosseous (FDI) muscle compared to neurotypical controls.
- Greater beta SMA-FDI connectivity at inpatient rehabilitation admission was associated with poorer motor recovery, although CMC did not significantly change during the inpatient rehabilitation stay.

**What are the implications of the main findings?**

- Beta SMA–FDI CMC may serve as a biomarker of neural injury and motor function early after stroke.
- The relationship between early CMC and motor recovery supports its potential prognostic value; however, the lack of change during inpatient rehabilitation highlights the need for longer-term longitudinal studies to clarify its utility as a biomarker of stroke recovery.

**Abstract:**

Background: Assessing cortical and muscle activity simultaneously during task performance may inform motor function post-stroke. This study evaluated brain–muscle functional connectivity (corticomuscular coherence, CMC) in early stroke recovery. Methods: Individuals with stroke in an inpatient rehabilitation facility (IRF) completed motor assessments and simultaneous electroencephalography (EEG) and electromyography (EMG) recordings during a grip task at IRF admission and discharge. Beta (20–30 Hz) CMC was measured between EEG electrodes overlying the primary motor cortex (M1) and supplementary motor area (SMA) and EMG leads overlying the first dorsal interosseous (FDI). Neurotypical controls completed identical EEG/EMG recordings. Correlational analyses were performed to ascertain CMC and motor assessment associations. CMC differences by Group (Stroke vs. Controls), Time (Admission vs. Discharge), and Extremity (Affected/Dominant vs. Less Affected/Non-Dominant) were estimated using mixed-effects linear models. Results: Thirty individuals with stroke (14 females, mean age 67.0 ± 9.8 years, 10.4 ± 3.5 days post-stroke) and 17 controls (8 females, mean age 75.3 ± 13 years) participated. Individuals with stroke exhibited reduced beta CMC between SMA and affected FDI (F_(1,36.1)_ = 5.73, *p* = 0.02, Cohen’s *f* = 0.40) compared to controls, with lower CMC involving the affected vs. less affected extremity (F_(1,73.0)_ = 5.72, *p* = 0.01, Cohen’s *f* = 0.28). Greater beta SMA–FDI CMC at admission related to poorer motor recovery (ρ = −0.59, *p* = 0.01). Group and Extremity CMC differences were not observed over time, nor were there changes in affected extremity CMC from admission to discharge. Conclusions: Beta SMA–FDI CMC is a marker of neural injury, exhibiting extremity-specific differences early post-stroke. While beta SMA–FDI CMC correlated with motor recovery, the absence of change over time during hospitalization necessitates longitudinal assessments to clarify its trajectory alongside recovery.

## 1. Introduction

Disruptions in neural network connectivity following stroke [1] contribute to deficits across multiple functional domains [2]. Functional connectivity, reflecting the correlation of spontaneous activity between distinct brain regions [3], has revealed that both inter- and intrahemispheric connections between primary and secondary motor areas inform post-stroke motor function and recovery [4,5,6]. Disruptions in these functional connections results in diminished neural drive to descending motor output pathways such as the corticospinal tract (CST) [7], which may further exacerbate motor system dysfunction. Structural measures of CST injury and integrity, which characterize the extent and quality of descending drive, are well-established biomarkers of post-stroke motor function and recovery [8,9,10]. Yet, these structural indices may not fully capture the complexity of post-stroke motor recovery involving neural network reorganization. Corticomuscular coherence (CMC), a measure of functional connectivity based on synchronous oscillatory activity between cortical and muscle sources during movement, represents communication between corticospinal projections and motor units via the CST [11,12,13]. By integrating information from both upstream cortical control and downstream muscle function, CMC has the potential to capture novel wisdom as a more comprehensive stroke recovery biomarker.

Seminal work by Conway et al. analyzed coherence between the sensorimotor cortex and the first dorsal interosseous muscle during isometric contractions in unimpaired individuals and observed synchronized cortical activity coupled with motor output, indicating that cortical neuronal activity facilitated motor unit synchronization [14]. Subsequent work has substantiated these initial findings, demonstrating coherence between beta (13–35 Hz) sensorimotor cortical oscillations and contralateral muscle activity during voluntary motor tasks [15,16,17]. Collectively, this work and others [18,19,20] suggests that CMC reflects brain–muscle communication whereby descending signals from sensorimotor cortical areas are transmitted downstream through the CST to the spinal cord to eventually drive motor neuron activity and facilitate movement execution. The magnitude of beta CMC increases from childhood to early adulthood, likely reflecting sensorimotor system maturation [21,22,23]. Beta-band CMC in adults is typically most prominent during steady voluntary muscle contractions, reflecting the precise synchronization between cortical and muscle activity that supports fine motor control [24,25] and greater motor precision [26,27].

In stroke, where CST integrity and motor control are often compromised, several studies have reported reduced beta CMC between specific cortical motor areas and upper-extremity musculature [28,29] across both acute [30,31] and chronic recovery timeframes [12]. Although some evidence indicates gradual increases in beta CMC between cortical motor areas and affected extremity musculature during early motor recovery [30,32], one study found no significant changes during the initial weeks post-stroke [31]. Furthermore, prior work has also identified a more dispersed distribution of peak CMC overlying the contralesional hemisphere when compared to unimpaired individuals, suggesting a reorganization of sensorimotor function [30,33]. Collectively, these studies highlight the utility of CMC as a measure of motor function and recovery post-stroke. However, most studies examined CMC during discrete movements such as elbow flexion [12,28], wrist extension [12,29,30], finger abduction [32], and pinch tasks [31]. While informative, these tasks have limited ecological validity and may not fully capture functionally relevant corticomuscular connections that underlie goal-directed movements essential for post-stroke motor function and recovery.

In this study, we sought to address the limited evidence regarding CMC during functionally relevant motor tasks after stroke by examining CMC during an isometric goal-directed grip task. We collected multiple CMC measurements during early post-stroke hospitalization at an inpatient rehabilitation facility (IRF) and included an unimpaired neurotypical cohort to examine the effects of neurological injury. Expanding on prior work that demonstrated reduced beta CMC between affected primary motor cortex (M1) and upper-extremity musculature post-stroke [12,30], we measured beta CMC between electrodes overlying both primary and secondary cortical motor areas, including M1, supplementary motor area, premotor and parietal cortices in both ipsi- and contralesional hemispheres and bilateral upper-extremity muscles (biceps, flexor and extensor digitorum and first dorsal interosseous) during an isometric, goal-directed grip task. We hypothesized that at the time of IRF admission, individuals with stroke would exhibit reduced beta CMC between ipsilesional primary and secondary motor regions and affected extremity compared to controls. We anticipated increases in beta CMC during IRF hospitalization, mapping to motor recovery, such that between-group CMC differences would not persist near the time of IRF discharge.

## 2. Methods

### 2.1. Participants

We recruited individuals aged 18 years and older with radiologically confirmed unilateral cortical and/or subcortical stroke (ischemic or hemorrhagic) hospitalized in an IRF. Exclusion criteria included active neurological or psychiatric disorders, conditions other than stroke affecting paretic upper extremity function, severe communication or cognitive impairments that would limit study participation, limited English proficiency, concurrent enrollment in another interventional study, contraindications to magnetic resonance imaging (MRI) or electroencephalography (EEG), prior stroke history, or bilateral hemispheric involvement. We also enrolled a control cohort of unimpaired right-handed individuals without a history of stroke via study flyers and mass email recruitment. All participants provided written consent according to study procedures approved by the Institutional Review Board at the University of North Carolina, Chapel Hill. This manuscript encompasses a cohort that was previously published [34,35,36].

Individuals with stroke completed two research visits that occurred around IRF admission and IRF discharge. During these visits, participants completed EEG and EMG recordings during a sub-maximal isometric grip task and an assessment battery evaluating motor impairment (Upper-Extremity Fugl-Meyer; UEFM) [37] and function (Action Research Arm Test; ARAT) [38]. During their IRF stay, individuals with stroke also completed a single MRI scan. Motor recovery was assessed by computing UEFM and ARAT Change_Realized_ scores during IRF stay. This was done by normalizing their assessment score change (Discharge−Admission) to recovery potential (Maximum Score−Admission) [8]. We used the Change_Realized_ score rather than raw change scores to account for variability in baseline impairment severity and recovery relative to initial injury. Unlike raw change scores, which may overestimate recovery in individuals with greater residual recovery potential, Change_Realized_ reflects improvement relative to the maximum possible recovery available to each participant at admission. Scores range from 0 to 1, with values closer to 1 indicating that participants have made significant progress toward their recovery potential. The control cohort completed a single research visit consisting of identical EEG and EMG recordings.

### 2.2. EEG and EMG Acquisition

Participants wore a dense array, 256-electrode EEG Hydrocel net (Electrical Geodesics, Inc., Eugene, OR, USA). Surface EMG electrodes were affixed to bilateral extensor and flexor digitorum, first dorsal interossei, and biceps brachii muscles using established anatomical landmarks corresponding to regions commonly identified with musculoskeletal ultrasound assessments. During EEG and EMG recordings, participants performed a goal-directed submaximal isometric grip task using a hand-held dynamometer with a custom Arduino circuit board. Participants were randomized so that half performed the task with their affected/dominant extremity first, followed by their less affected/non-dominant extremity. Prior to recording, we determined participants’ average maximal voluntary isometric force for each extremity across three trials and determined 20% of their average maximal voluntary isometric force for each extremity. During EEG and EMG recordings, participants performed isometric squeezing motions at 20% of their average maximal voluntary force output using a visual target that provided real-time feedback on the isometric force output. We chose this force value since prior research indicated that 20–50% of maximal force output corresponds with functional activities [39]. Additionally, this value allows for the collection of multiple trials while minimizing participant fatigue.

We selected an isometric grip task because gripping is integral to many activities of daily living, including object manipulation, lifting, carrying, and self-care tasks, making it a functionally meaningful upper extremity behavior after stroke. Compared with isolated single-joint movements, gripping involves coordinated activation across multiple upper extremity muscle groups and is typically more feasible to perform compared to fine motor tasks for individuals early post-stroke. Although the task was isometric, participants were required to continuously modulate and sustain force output, which necessitated ongoing visuomotor integration, force regulation, and motor execution beyond the simple discrete movements commonly examined in prior studies [20,28]. Participants performed 5 practice trials before completing 2 blocks of 20 trials per extremity with a 1 min break between blocks. Each trial lasted approximately 5 s with inter-trial intervals ranging from 7 to 15 s to prevent habituation. Participants maintained a comfortable sitting position throughout the experiment with their forearm and wrist positioned to best accommodate task requirements. Data were sampled at 1000 Hz using a Net Amp 400 amplifier and Net Station 5.4.2 software (Electrical Geodesics, Inc., Eugene, OR, USA).

### 2.3. EEG and EMG Preprocessing Steps and Measures

Raw EEG data were preprocessed using methods consistent with those described in prior published work [34]. EMG data were transferred to MATLAB 2017b for offline processing that involved bandpass filtering from 10 to 50 Hz and 70 to 100 Hz. Unrectified data were passed through a Hilbert transform to obtain the signal envelope before rectification. EEG and EMG data were concatenated with CMC trial windows defined as 1000 ms before and 4000 ms after stimulus onset. CMC computation was performed using the Fieldtrip toolbox [40]. Data were flipped so that the left hemisphere and right upper extremity corresponded to the ipsilesional hemisphere and affected extremity for all participants with stroke.

In this study, we measured CMC between ipsi- and contralesional primary and secondary motor brain areas and bilateral upper extremity muscles across delta (1–3 Hz), low beta (13–19 Hz), and high beta (20–30 Hz) frequency bands, which inherently yield numerous possible brain–muscle combinations. To increase specificity and reduce the number of multiple comparisons, we focused our analysis on CMC measurements between electrodes overlying the ipsilesional M1 and the supplementary motor area (SMA) and EMG electrodes overlying the affected first dorsal interosseous (FDI). We selected M1 due to its role in movement execution [41,42] as well as previous work that indicated reduced CMC between M1 and upper-extremity musculature post-stroke [28,29,31,43]. We included the SMA given its role in motor planning and execution of goal-direction behavior [44], and prior work depicting post-stroke SMA activation during a grip task [45,46]. We focused on the FDI due to its role in stabilizing and facilitating grip precision, factors essential for an isometric goal-directed grip task. Our primary CMC analyses included computing average coherence value across trials for the high beta (20–30 Hz) frequency band given the prominence of high beta oscillatory activity during isometric voluntary muscle contractions that require sustained visually guided grip force control, involving continuous sensorimotor integration and force regulation [14]. Additionally, high beta activity has been implicated in goal-directed motor control [47,48] and previous studies have consistently reported reduced beta CMC following a stroke [11,29,43]. CMC values are reported here as the analog of the squared correlation coefficient between EEG and EMG signals [13]. Coherence is calculated with respect to the cross-spectrum density of signals S1 and S2, for each frequency of interest (f) (P_S1,S2_(f)) and the auto-spectrum densities of S1 and S2 for each frequency of interest (P_S1_(f) and P_S2_(f)), as noted in the following equation [13].

$${Coh}_{S1,S2}\left(f\right)=\frac{{\left|P_{S1,S2}\left(f\right)\right|}^{2}}{\left|P_{S1}\left(f\right)\right|\times \left|P_{S2}\left(f\right)\right|}$$

Values range from 0 (indicating random amplitude ratios and phase differences between signals across time) to 1 (consistent amplitude ratios and phase differences between two signals across time). To further characterize CMC differences based on Group (Stroke vs. Controls) and Time (IRF Admission vs. Discharge), topographic plots depicting CMC magnitude across the scalp surface were generated and visually inspected.

### 2.4. Structural Imaging and Injury Quantification

Structural MRIs were acquired on either a 3-Tesla Siemens MAGNETOM TrioTim Syngo or Skyra scanner or a 1.5-Tesla Siemens MAGNETOM Aera scanner (all systems from Siemens Healthcare, Erlangen, Germany). Participants with stroke completed both a structural T1 sequence (repetition time = 2300 ms, echo time = 2.91–3.26 ms, 160 slices, 1 mm^3^ isotropic voxel) and a T2-weighted fluid-attenuated inversion recovery sequence (repetition time = 9000 ms, echo time = 115 ms, 31 slices, voxel size = 0.9 × 0.9 × 5 mm^3^). We computed lesion volume and percent injury to CST using previously validated protocols in stroke [9].

### 2.5. Statistical Analysis

A priori sample size estimation was performed based on a previously published study by Krauth et al. that recruited five individuals with stroke and seven unimpaired right-handed controls [30]. In that study, individuals with stroke underwent three sessions of EEG and EMG recording at approximately two weeks, seven weeks, and one-year post-stroke. Beta (12–30 Hz) CMC between the M1 and wrist extensors was assessed. Motor impairment was evaluated using the UEFM. Based on the reported change in beta CMC over time, a total sample size of 34 individuals (17 per group) was required to achieve 80% power at an alpha level of 0.05.

Statistical analyses were conducted in JMP Pro17 (SAS Institute Inc., Cary, NC, USA). A series of mixed-effects linear models were employed to evaluate CMC differences with respect to Group (Stroke vs. Controls), Time (Admission vs. Discharge), and Extremity (Affected/Dominant vs. Less Affected/Non-Dominant) effects. As CMC reflects communication between brain and muscle via the CST [13] and previous work indicates a decline in CMC with aging [49], both Age and Percent Injury to CST were included in models as fixed-effect covariates to adjust for confounding. Each participant served as a random intercept to model within-subject correlation. Model construction proceeded in stages with the addition of fixed effects, followed by interactions between fixed effects and then the random effect. Model fit was assessed using the Akaike Information Criterion (AIC) and Bayesian Information Criterion (BIC) estimators of prediction error, with lower values indicating better model fit. The model with the lowest AIC and BIC was selected for interpretation. Both metrics were included to provide complementary assessments of model performance, as AIC emphasizes predictive accuracy whereas BIC imposes a greater penalty for model complexity. Post hoc analyses involved pairwise comparisons using Tukey’s honestly significant difference test, with statistical significance set at an alpha value of 0.05. Model assumptions of normal distributions of residuals and homoskedasticity were confirmed with Shapiro–Wilk and Levene tests, respectively. Correlation coefficients were computed to determine associations between CMC measurements and motor recovery (Change_Realized_ values for UEFM and ARAT). To further characterize CMC, we performed exploratory topographical analyses comparing CMC values between IRF admission and discharge. At each electrode, paired-sample t-tests were conducted to evaluate differences in beta CMC with the affected FDI. To control for the inflation of Type I error across multiple electrodes, *p*-values were adjusted using the Benjamini–Hochberg false discovery rate (FDR) procedure (α = 0.05). Electrodes meeting FDR-corrected significance criteria were considered to exhibit statistically significant changes in CMC across time. For visualization purposes, topographic maps of z-transformed beta CMC were generated for admission, discharge, and the difference between time points.

## 3. Results

We enrolled 30 individuals with stroke (14 females, 67.0 ± 9.8 years of age, 10.4 ± 3.5 days post-stroke, length of IRF stay: 15.5 ± 6.2 days) and 17 control participants (8 females, 75.3 ± 13 years of age). Participant demographics and behavioral assessment scores are depicted in Table 1 and described in detail elsewhere [34,35,36]. Of the 30 participants, 21 participants completed the task with their affected extremity at admission and 24 participants at discharge. Nine participants who were unable to complete the task at admission demonstrated severe motor impairment (UEFM: 5.3 ± 3.8, range 2–12; ARAT: 0; NIHSS total: 8.7 ± 4.3, range 3–17; NIHSS motor: 5.3 ± 1.8, range 3–8). At discharge, six participants did not complete the task (two missed the second assessment and four remained unable to perform the task), and these participants also exhibited substantial impairment (UEFM: 15.5 ± 21.0; ARAT: 9.3 ± 18.5; NIHSS total: 6.7 ± 5.6; NIHSS motor: 4.5 ± 3.0). Therefore, analyses involving the affected-extremity task predominantly reflect participants with sufficient residual motor function to perform the task. All 30 participants performed the task with their less-affected extremity, and data were retained from 26 and 27 participants at admission and discharge time points, respectively. For controls, data collected from both extremities were retained for 14 participants. Due to the presence of motion artifact in the EEG recordings, data from four individuals with stroke and three controls were partially or completely discarded. Excessive motion artifact was considered unsuitable for analysis because it can introduce non-neural EEG activity (i.e., noise) and distort spectral estimates within the frequency range used to calculate CMC, potentially resulting in spurious coherence measurements that do not reflect true corticomuscular interactions. All available data were included in the linear mixed-effects models.

### 3.1. CMC Between-Group Differences Across Time

To evaluate differences in beta M1–FDI and SMA–FDI CMC in individuals with stroke and controls across time, we used a linear mixed-effects model with fixed effects of Group (Stroke vs. Control), Time (Admission vs. Discharge), Age, and a Group x Time interaction. The inclusion of Group x Time interaction was driven by our hypothesis that individuals with stroke may exhibit changes in CMC during the post-stroke recovery period. Control participants completed a single visit as we assumed that CMC would remain stable over time in neurologically intact individuals. Accordingly, control data were treated as time-invariant. This approach allowed controls to be included in the model as a reference group while avoiding estimation of a Group x Time interaction for controls, thereby preventing non-positivity and aligning with the assumption of no systematic change over time in the control group. Additionally, given the broad participant age range in both groups (Table 1) and a significant age difference between groups (stroke: 67.0 ± 9.8 years, control: 75.3 ± 13 years; t_(15)_ = 2.69, *p* = 0.01), we included Age in the model.

For beta M1–FDI CMC, no significant main effects of Group (F_(1,36.9)_ = 0.29, *p* = 0.59, Cohen’s *f* = 0.09), Time (F_(1,34.4)_ = 1.35, *p* = 0.25, Cohen’s *f* = 0.20), or Group x Time interaction effect (F_(1,34.4)_ = 1.35, *p* = 0.25, Cohen’s *f* = 0.20) were observed.

For beta SMA–FDI CMC, there was a significant main effect of Group (F_(1,36.1)_ = 5.73, *p* = 0.02, Cohen’s *f* = 0.40), with no effects of Time (F_(1,32.2)_ = 0.08, *p* = 0.77, Cohen’s *f* = 0.05) or the Group x Time interaction (F_(1,32.2)_ = 0.08, *p* = 0.77, Cohen’s *f* = 0.09). These findings indicate reduced beta SMA–FDI CMC in the stroke group relative to controls.

### 3.2. CMC Within-Group Differences over Time

We assessed changes in affected extremity CMC during hospitalization using models including Time, Age, and Percent Injury to CST. No significant effect of Time was observed for either M1–FDI CMC (F_(1,24.7)_ = 1.73, *p* = 0.19, Cohen’s *f* = 0.27) or SMA–FDI CMC (F_(1,23.4)_ = 0.0009, *p* = 0.97, Cohen’s *f* = 0.01).

### 3.3. CMC Differences Between Extremities

We assessed differences in CMC between extremities using separate mixed-effects models for controls and stroke groups.

**Figure 1:**
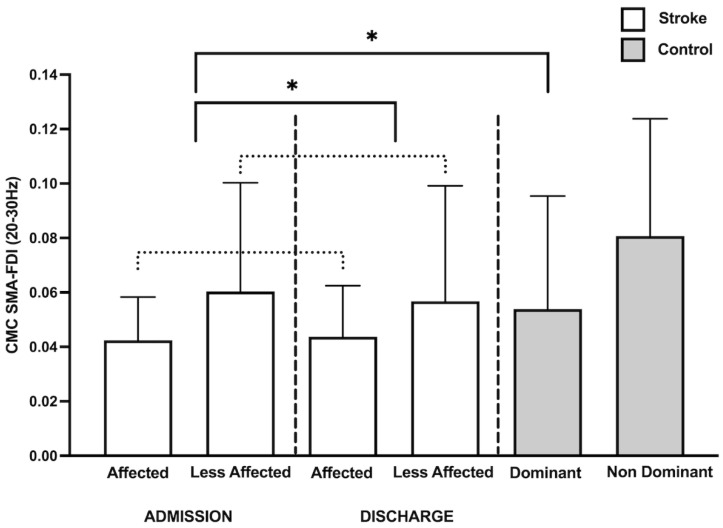
Individuals with stroke exhibited overall (collapsed across visits) reduced beta corticomuscular coherence (CMC) between supplementary motor area (SMA) and first dorsal interossei (FDI) in the affected extremity compared to the dominant extremity of controls (F_(1,36.1)_ = 5.73, *p* = 0.02). Within the stroke group, overall (collapsed across visits) SMA–FDI beta CMC was lower on the affected side compared to the less affected side Extremity (F_(1,73.0)_ = 5.72, *p* = 0.01). Dotted lines indicate that the data was collapsed across visits. * indicates *p* < 0.05.

In controls, models included fixed effects of Age and Extremity. M1–FDI CMC did not differ between extremities (F_(1,40.9)_ = 1.18, *p* = 0.28, Cohen’s *f* = 0.17). In contrast, SMA–FDI CMC showed a significant effect of Extremity (F_(1,43.6)_ = 8.59, *p* = 0.005, Cohen’s *f* = 0.45), with lower CMC in the dominant vs. non-dominant limb (Figure 1).

In the stroke group, models included Time, Extremity, Age, Percent Injury to CST, and a Time x Extremity interaction, consistent with our hypothesis that changes in CMC from the affected extremity would occur during the post-stroke recovery period. For M1–FDI CMC, Time (F_(1,75.0)_ = 0.04, *p* = 0.82, Cohen’s *f* = 0.02), Extremity (F_(1,73.3)_ = 0.29, *p* = 0.58, Cohen’s *f* = 0.06), and Time x Extremity (F_(1,68.9)_ = 3.84, *p* = 0.06, Cohen’s *f* = 0.24) effects were not significant. For SMA–FDI CMC, there was a significant main effect of Extremity (F_(1,73.0)_ = 5.72, *p* = 0.01, Cohen’s *f* = 0.28), indicating lower CMC in the affected vs. less-affected limb (Figure 1) with no effects of Time (F_(1,74.6)_ = 0.02, *p* = 0.88, Cohen’s *f* = 0.02) or the interaction (F_(1,68.5)_ = 0.05, *p* = 0.81, Cohen’s *f* = 0.03).

### 3.4. Affected Extremity CMC and Motor Recovery

**Figure 2:**
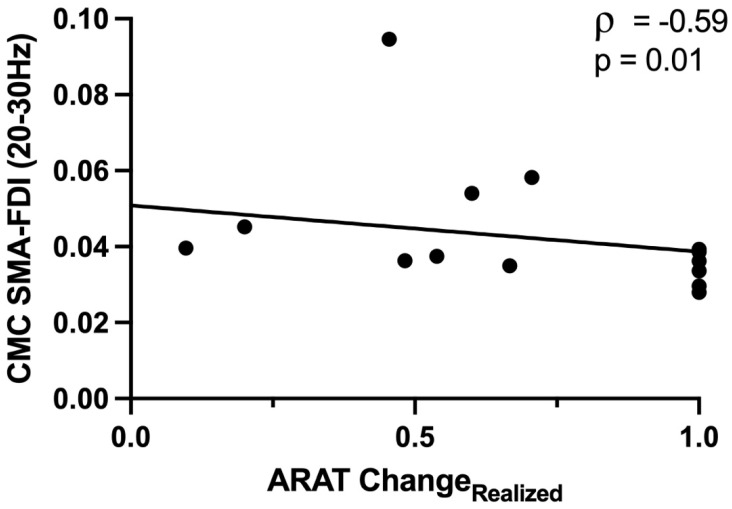
Greater beta corticomuscular coherence (CMC) between supplementary motor area (SMA) and first dorsal interosseous (FDI) at admission was associated with less motor recovery during hospitalization, as measured by Action Research Arm Test Change_Realized_ scores.

Participants showed significant and clinically meaningful [50,51] gains in motor recovery (UEFM change = 6.4 ± 9.6 points; ARAT change = 5.3 ± 9.2 points) during IRF hospitalization. Beta M1–FDI CMC involving the affected extremity at admission did not correlate with motor recovery (UEFM Change_Realized_: ρ = 0.27, *p* = 0.29; ARAT Change_Realized_: ρ = −0.25, *p* = 0.35). Beta SMA–FDI CMC at admission also did not relate to motor impairment change (UEFM Change_Realized_: ρ = −0.46, *p* = 0.06). However, greater beta SMA–FDI CMC at admission was associated with poorer motor recovery (ARAT Change_Realized_ scores: ρ = −0.59, *p* = 0.01; Figure 2).

### 3.5. Topographical Observations

**Figure 3:**
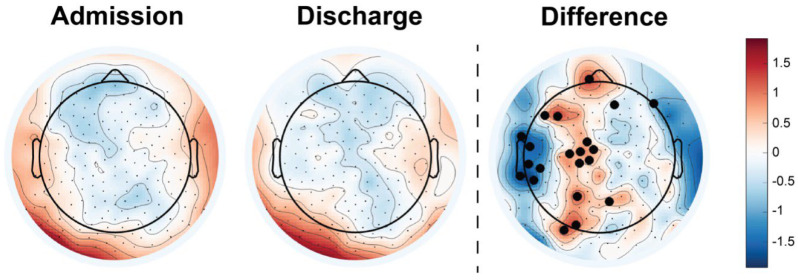
Topographical plots demonstrating the z-transformed beta (20–30 Hz) coherence across the scalp with the first dorsal interosseous in individuals with stroke near hospitalization admission and discharge. Change in beta CMC during hospitalization is plotted with significant electrodes denoted in black (p_corrected_ < 0.05). Red areas on the topographical plots correspond to higher beta CMC values between EEG electrodes and the affected FDI muscle during task performance.

To examine spatial changes in beta CMC beyond predefined regions, we generated whole-brain topographic maps to visualize the distribution of z-transformed beta CMC across the scalp at IRF admission and discharge (Figure 3, left and middle), as well as the difference between time points (Figure 3, right). These maps suggested a redistribution of beta CMC over hospitalization, characterized by increases over midline frontal electrodes overlying SMA and central/frontal regions overlying ipsilesional M1 and dorsolateral frontal regions, and decreases over ipsilesional temporal areas. Electrode-wise paired t-tests supported these patterns, revealing significant clusters (≥3 adjacent electrodes) with increased CMC over midline frontal regions and decreased CMC over ipsilesional temporal areas (Figure 3, right).

## 4. Discussion

This study evaluated the role of beta CMC during an isometric goal-directed grip task in individuals with subacute stroke. Our findings partially supported our hypothesis as individuals with stroke exhibited reduced beta CMC in the affected extremity compared to the dominant extremity of controls, specifically between the SMA and FDI. In stroke, beta CMC between SMA and FDI was lower on the affected vs. less-affected side. While no significant group or extremity differences were observed at specific time points, and no changes in affected extremity CMC were identified from admission to discharge, CMC at IRF admission related to motor recovery. Collectively, these findings provide preliminary support for CMC as a potential biomarker of early stroke motor system function.

To capture neural dynamics contributing to motor recovery, we measured CMC from EEG electrodes overlying both M1 and SMA. Contrary to our hypothesis, beta CMC between M1 and FDI—a proxy of CST transmission—was not significantly altered. Instead, our findings suggest that beta CMC between SMA and FDI may be more sensitive to stroke-related changes compared to the predominant M1-upper extremity functional connections that have been the primary focus in previous studies. This distinction may be attributed to task variations and complexity. Previous studies mainly evaluated CMC during motor tasks like wrist extension or finger abduction movements [12,30,31,32], which involve isolated joint movements and largely engage M1. While these tasks have demonstrated utility in the measurement of CMC, they may not fully convey the motor coordination required for functional tasks. In contrast, we employed an isometric goal-directed grip task that we assert is relevant to daily activities like grasping and holding objects. The heightened SMA–FDI CMC in our study aligns with the well-documented role of SMA in goal-directed behavior [52]. The SMA plays a crucial role in regulating grip force, motor timing, and performance monitoring– key elements in executing this isometric goal-directed grip task [44,45]. The continuous monitoring and adjustment of motor output, as required by an isometric grip task, may further accentuate the involvement of the SMA, making SMA–FDI CMC a more sensitive indicator of functional changes. From a neurophysiological perspective, reduced beta SMA-FDI CMC observed in this study may indicate impaired transmission or synchronization of motor commands through corticospinal pathways, resulting in less efficient coupling between cortical motor planning processes and muscle activation [36]. Prior research in young, unimpaired adults indicates that corticospinal projections from the SMA are recruited during tasks requiring precise force control, further supporting the role of SMA [46]. Our findings underscore the importance of selecting functionally relevant motor tasks during CMC measurement that better reflect real-life motor demands and identify specific brain–muscle connections that could serve as reliable biomarkers of motor recovery post-stroke. Additionally, because lesion size and lesion location were not accounted for in our analyses, heterogeneity across participants may have further obscured M1–FDI coherence patterns at the group level.

Individuals with stroke also exhibited reduced beta CMC between the SMA and FDI in the affected extremity compared to the dominant extremity of controls. This is potentially due to impaired motor network connectivity post-stroke, which may impact functional communication between the SMA and peripheral muscles. At a systems level, reduced CMC may indicate disrupted coupling between cortical beta oscillations and downstream spinal motor neuron activity. This disruption likely impacts motor control, including the ability to maintain stable force output required for effective execution of goal-directed tasks in individuals post-stroke. Reduced SMA–FDI CMC may therefore represent a clinically relevant marker of impaired descending motor drive to distal hand muscles which are routinely targeted in both clinical interventions and rehabilitation strategies post-stroke [53]. Additionally, our findings indicate that individuals with stroke exhibit lower CMC between the SMA and FDI on the affected side compared to the less-affected side, further reinforcing the notion of disrupted corticomuscular connectivity following stroke. Yet, we observed no significant changes in affected extremity CMC from admission to discharge. These results align with previous findings by Larsen et al., who reported no significant CMC alterations from the acute to sub-acute stages of stroke recovery (3 to 38 days post-stroke) [31]. Studies showing changes in CMC over time have mainly focused on alterations from acute to chronic stroke stages [30,32], suggesting that early assessment windows may not capture robust alterations in functional connectivity between brain and muscle. This lack of change may be attributed to the time-sensitive nature of motor recovery. For instance, the Critical Period After Stroke Study (CPASS) identified a sensitive recovery window of 60–90 days post-stroke [54]. Rodent stroke models support this timeline, showing that dendritic branching peaks 2–3 weeks post-stroke, followed by behavior-dependent pruning [55,56]. Furthermore, Murphy and Corbett proposed that homeostatic-like neuroplastic mechanisms driving the restoration of connectivity predominantly occur during the initial 1–4 weeks post-stroke in rodents [57]. Additionally, muscle recovery in individuals with stroke also peaks within the first 3 months [58,59]. Muscle recovery dynamics may thus impact CMC measurements. Although participants demonstrated behavioral improvements during IRF stay, the relatively short assessment window (5 to 14 days post-stroke) may be insufficient for capturing significant connectivity change between cortical and muscular sources. It could be postulated that behavioral improvements may have emerged from early neural reorganization, compensatory network recruitment, or rehabilitation-driven motor strategies before measurable changes in synchronized corticospinal–muscular coupling were established. Additionally, peripheral muscular adaptations and restoration of stable corticospinal communication may evolve on a different timescale than observable functional gains, potentially contributing to the absence of significant longitudinal CMC changes despite improvements in UEFM and ARAT scores.

The incorporation of topographical plots in this work provided valuable visualization of CMC changes beyond the ipsilesional M1 and SMA regions, illustrating spatial shifts in CMC during IRF hospitalization. At IRF admission, individuals post-stroke showed reduced CMC across sensorimotor areas, consistent with early disruption of motor network integration. By discharge, statistical testing identified significant decreases in CMC over the ipsilesional temporal regions alongside increases in the midline frontal regions encompassing SMA-related sites. The observed increases in SMA-related coherence align with the role of SMA in coordinating goal-directed actions and regulating grip force, highlighting its potential compensatory role during early rehabilitation. Together, these findings extend focal SMA–FDI effects by demonstrating that stroke recovery is accompanied by broader topographical shifts in CMC, emphasizing the value of whole-brain approaches for capturing network-level reorganization.

The absence of significant extremity differences in CMC at specific time points further suggests that the time frame between admission and discharge in our study may not have been sufficient to capture alterations in CMC. Interestingly, in the control group, we observed higher CMC between SMA and FDI in the non-dominant extremity when compared to the dominant extremity. This is consistent with prior work by Beck et al., which showed increased cortical engagement in the less-skilled extremity to maintain motor control [23]. Our results suggest that this compensatory mechanism may be altered in individuals post-stroke. Although the affected extremity is also less-skilled, it is additionally influenced by disruptions stemming from CST injury, which may limit the capacity to increase CMC as a compensatory response. Consistent with this interpretation, we observed reduced CMC between the SMA and FDI in the affected extremity, suggesting impaired descending motor drive rather than a skill-related compensatory adaptation. Lastly, the observed differences in CMC between dominant and non-dominant extremities in controls may prompt the consideration of pre-stroke hand dominance and hemisphere of injury when interpreting CMC in individuals with stroke [60].

Lastly, we investigated the relationship between CMC and motor recovery as measured by Change_Realized_ values for UEFM and ARAT. The use of Change_Realized_ scores allowed recovery to be interpreted relative to baseline impairment severity, which was important given the heterogeneity of motor deficits in our cohort. This normalization accounts for individual recovery potential and reduces bias related to ceiling effects in participants with milder impairment. Our findings revealed a negative association between SMA–FDI CMC in the affected extremity at admission and motor recovery, as measured by Change_Realized_ ARAT scores. These results suggest that individuals with greater SMA–FDI CMC at IRF admission experience less motor recovery. This finding appears to contradict our original hypothesis that reduced beta CMC would be associated with poorer motor function and recovery. Importantly, this finding should be interpreted in the context of our group-level results demonstrating reduced SMA–FDI CMC in individuals with stroke relative to controls. At the group level, lower CMC likely reflects disruption of normal corticomuscular communication following stroke. However, within the stroke cohort, variability in SMA–FDI CMC may reflect differences in the extent of neural injury and compensatory network recruitment. Thus, while stroke is generally characterized by reduced CMC, relatively greater SMA–FDI CMC among individuals with stroke may not necessarily indicate a more intact motor system. When considered alongside our prior work that indicated increased CMC with greater CST injury [36], this finding implies that increased CMC early after stroke may reflect a compensatory response to greater CST damage that may not necessarily equate to better recovery outcomes and could therefore be maladaptive. Under this framework, individuals exhibiting greater SMA–FDI CMC shortly after stroke may be those with more substantial CST injury and consequently reduced recovery potential. This interpretation aligns with previously published work that indicated that in patients with damaged CST, stronger task-related activity in the SMA was associated with worse residual motor function [45]. An alternative interpretation is that individuals with relatively preserved SMA–FDI CMC at admission may have greater baseline motor function, and therefore have a reduced recovery window, even when accounting for baseline performance using Change_Realized_ scores. These interpretations are not mutually exclusive and may reflect different mechanisms operating across the spectrum of CST injury severity. Together, these findings suggest that the functional relevance of post-stroke brain–muscle connectivity may depend on the extent of CST injury. Future studies should distinguish adaptive from maladaptive connectivity patterns by stratifying participants according to CST injury severity. In particular, comparing individuals with mild-to-moderate versus severe CST injury may help clarify how CMC adaptations vary with injury burden, as broadly assuming increased CMC to be beneficial for motor recovery likely oversimplifies the underlying recovery mechanisms.

We acknowledge limitations in this work. Individuals with severe hemiparesis were not able to complete the grip task using their affected extremity, which accentuates barriers related to task demand and the sensitivity of our equipment to acquire force output exerted by those participants. Our findings, therefore, may not generalize to those with severe hemiparesis post-stroke. We did not exclude participants based on lesion location and extent of damage to cortical regions of interest, which may impact EEG signal interpretation and CMC values, as different lesion profiles may variably affect neural connectivity. Further, we did not account for pre-stroke hand dominance and length of IRF stay, which could have confounded the interpretation of our results. Lastly, the sample included in this study exhibited a potential ceiling effect on ARAT, which may have constrained the observed adaptive functional connectivity patterns post-stroke. Establishing whether early changes in CMC reflect adaptive or maladaptive processes will be a critical next step for translating this measure into a clinically relevant biomarker.

## 5. Conclusions

This study determined the potential of CMC as a biomarker of post-stroke motor function and recovery using a functionally relevant isometric grip task. In the context of this task, we identified beta CMC between SMA- and FDI-differentiated individuals with stroke from neurotypical controls and was associated with motor recovery, suggesting that SMA–FDI connectivity captures clinically relevant aspects of post-stroke motor function. However, the absence of time-specific changes in CMC during IRF hospitalization indicates that CMC may not be sufficiently sensitive to track short-term recovery over the early post-stroke period. Together, these findings support the potential utility of CMC as an indicator of motor system status after stroke, while highlighting the need for longer-term longitudinal studies to determine its value as a biomarker of stroke motor recovery.

## Figures and Tables

**Table 1:** Participant characteristics and behavioral assessment scores.

Descriptor	Sample Size	Mean (SD) Median [IQR]	Range
Sex *Stroke*			
Female	14	-	-
Male *Control* Female Male	16 8 9	-	-
Race, Ethnicity *Stroke*			
Black, non-Hispanic	6	-	-
White, non-Hispanic *Control* Black, non-Hispanic White, non-Hispanic Asian	24 16 1	-	-
Age (years) *Stroke* *Control*		67 (9.8) 75.3 (13)	51–85 48–95
Stroke Type			
Hemorrhagic	2	-	-
Ischemic	28	-	-
Lesioned Hemisphere			
Left	12	-	-
Right	18	-	-
Days Post-stroke to Enrollment		10.4 (3.5)	5–18
Length of IRF Stay		15.5 (6.2)	4–29
NIHSS (max = 42)		3 [1–6.2]	0–17
Lesion Volume (cc)		19.9 (35.9)	0.08–155.7
Percent Injury to CST		58.3 (40.6)	0–100
UEFM (max = 66)			
Visit 1		40.9 (25.1)	2–66
Visit 2	29	46.8 (23.6)	4–66
ARAT (max = 57)			
Visit 1		33.0 (23.7)	0–57
Visit 2	28	37.1 (24.1)	0–57
MoCA (max = 30) *Stroke*			
Visit 1	28	22.3 (4.2)	14–29
Visit 2	29	23.9 (5.4)	11–30
*Control*		25.8 (3.4)	20–30

## Data Availability

Anonymized data are available and may be accessed through UNC Dataverse at https://doi.org/10.15139/S3/KPJCQL.
